# A Composite-Type MEMS Pirani Gauge for Wide Range and High Accuracy

**DOI:** 10.3390/s23031276

**Published:** 2023-01-22

**Authors:** Shuo Chen, Liuhaodong Feng, Song Guo, Yucheng Ji, Shuwen Zeng, Xinlin Peng, Yang Xu, Tianbao Hu, Zhenyu Wu, Shinan Wang

**Affiliations:** 1 School of Microelectronics, Shanghai University, Shanghai 201899, China; 2 Shanghai Industrial µTechnology Research Institute, Shanghai 201899, China; 3 Shanghai Institute of Microsystem and Information Technology, Chinese Academy of Sciences, Shanghai 200031, China

**Keywords:** MEMS Pirani gauge, composite type, wide range, high accuracy, thermal conductivity, simulation by COMSOL Multiphysics

## Abstract

To achieve a wide range and high accuracy detection of the vacuum level, for example, in an encapsulated vacuum microcavity, a composite-type MEMS Pirani gauge has been designed and fabricated. The Pirani gauge consists of two gauges of different sizes connected in series, with one gauge having a larger heat-sensitive area and a larger air gap for extending the lower measurable limit of pressure (i.e., the high vacuum end) and the other gauge having a smaller heat-sensitive area and a smaller air gap for extending the upper measurable limit. The high-resistivity titanium metal was chosen as the thermistor; SiN_x_ was chosen as the dielectric layer, considering the factors relevant to simulation and manufacturing. By simulation using COMSOL Multiphysics and NI Multisim, a range of measurement of 2 × 10^−2^ to 2 × 10^5^ Pa and a sensitivity of 52.4 mV/lgPa were obtained in an N_2_ environment. The performance of the fabricated Pirani gauge was evaluated by using an in-house made vacuum test system. In the test, the actual points of measurement range from 6.6 × 10^−2^ to 1.12 × 10^5^ Pa, and the highest sensitivity is up to 457.6 mV/lgPa. The experimental results are better in the range of measurement, sensitivity, and accuracy than the simulation results. The Pirani gauge proposed in this study is simple in structure, easy to manufacture, and suitable for integration with other MEMS devices in a microcavity to monitor the vacuum level therein.

## 1. Introduction

Some sensors, especially MEMS (microelectromechanical system) sensors, need to work in a vacuum environment for long periods [1]. Such MEMS devices include MEMS gyroscopes and resonant accelerometers, where the vacuum environment reduces the air damping of the accelerometer vibration structure to improve the performance of devices [2,3]. The quality factor is an important parameter describing the vibration characteristics of a MEMS gyro-mechanical structure, which is strongly influenced by the gas pressure within a certain vacuum level [4]. Therefore, high vacuum requirements are imposed when the device is packaged. However, the vacuum environment will gradually deteriorate with time due to the outgassing effect [5], which can affect the performance of the device. To ensure that the sensor is working in a proper environment, it is desirable to monitor the vacuum level in the cavity in real-time. This requires a vacuum gauge integrated with the MEMS device in the same vacuum cavity and with enough measuring range, accuracy, and even sensitivity.

Vacuum gauges are used to measure air pressure using certain effects of gases at different air pressures. According to the vacuum gauge measuring principles, the main vacuum gauges are classified into three types:

- Mechanical performance: Bourdon gauges and film capacitor gauges;
- Gas dynamic effects: Pirani gauges and thermocouple gauges;
- Charged particle effect: Cathodic ionization gauges.

Compared with other vacuum gauges, Pirani gauges have relatively small sizes, simple structures, larger measurement ranges, and high resolution, so they are widely used for monitoring the vacuum level in tiny cavities [6].

In our previous work, we designed a thin-film getter-microheater unit to maintain the vacuum level inside a device cavity [7]. However, to ensure that the device is kept working at an optimum vacuum level environment, a vacuum gauge is desired to be integrated inside the cavity to monitor the vacuum level therein. On the other hand, a conventional Pirani gauge has the shortcomings of a relatively narrow range of measurement, low sensitivity, and low accuracy. We have designed a high-sensitivity Pirani gauge in previous work [8]. In addition, a prior work demonstrates that a composite-type Pirani gauge is promising for a vacuum detection sensor of a wide range: Two gauges of different sizes are connected in series, one with the larger thermal area being more sensitive in the lower-pressure range and the other more sensitive in the higher-pressure range [9]. In their work, the concept of a diode-type composite Pirani gauge is demonstrated [9]. Stimulated by the concept in Ref. [9], in this work, we propose a thermistor-type composite Pirani gauge that is compatible with the aforementioned getter-microheater unit [7].

## 2. Theoretical Background

The operation principle of a Pirani gauge is quite simple: in a closed space, e.g., a microcavity encapsulated by a thermistor, the number of molecules per unit volume varies according to the vacuum level therein. More gas molecules per unit volume at high vacuum levels will dissipate heat from the thermal area, resulting in different heat dissipation capacities in the thermal area of the thermistor. This causes the temperature of the thermistor to vary. Since the resistance of a thermistor is a function of temperature, the resistance value varies at different temperatures. The change in resistance can then be amplified by a post-processing circuit and converted to an output voltage value. Therefore, the vacuum level in the microcavity can be accurately monitored by reading the output electrical signal.

**Figure 1:**
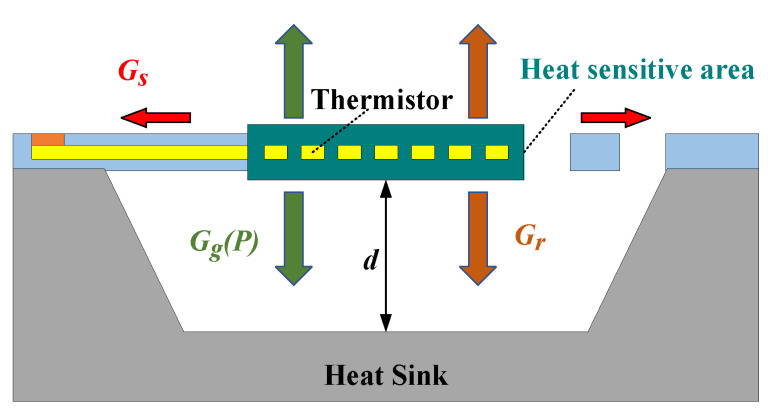
Thermal conductivity mechanism of the Pirani gauge.

When given an input voltage, the thermistor generates Joule heat due to the electrothermal effect. The generated total heat *Q_total_* equals the total heat lost *Q_loss_* when it reaches a self-heating balance at a certain temperature. It is well known that there are three mechanisms of heat transfer: heat conduction, heat convection, and heat radiation. In a closed microcavity, the effect of thermal convection can be neglected. Therefore, as shown in Figure 1, the thermal conductivity of this model includes the following:

1. Heat loss by solid conduction (*Q_s_*) through the cantilever structures supporting the heat-sensitive area with a thermal conductivity of *G_s_*;
2. Heat loss by gas conduction between the heat-sensitive area and the heat sink (*Q_g_(P)*) with a thermal conductivity of *G_g_(P)*;
3. Heat loss by thermal radiation *Q_r_* with a thermal conductivity of *G_r_*.

The thermal conduction can be simply expressed by Equations (1)–(3):

(1) $$Q_{total}=Q_{loss}=Q_{s}+Q_{g}\left(P\right)+Q_{r}$$

(2) $$Q_{total}=\left(T-T_{0}\right)\left(G_{s}+G_{g}\left(P\right)+G_{r}\right)$$

(3) $$G_{total}=G_{s}+G_{g}\left(P\right)+G_{r}$$

The thermal conductivity of solid conduction, *G_s_* depends on the length, width, and thickness of the cantilevers supporting the thermistor area and can be expressed as

(4) $$G_{s}=\lambda \frac{\omega t}{L}$$

where *λ* is the solid thermal conductivity, while *L*, *w*, and *t* are the length, width, and thickness of the supporting cantilevers, respectively.

The thermal conductivity of gas conduction, *G_g_*, is the most crucial point of the Pirani gauge since only the gas conduction is associated with the air pressure of the microcavity, and

(5) $$G_{g}\left(P\right)=k\frac{A}{d}$$

where *d* is the air gap between the thermal area and the heat sink, *A* the size of the thermal area, and *k* is the thermal conductivity of gas conduction.

The thermal radiation can be calculated using the Stefan–Boltzmann law and expressed as:

(6) $$Q_{r}=\sigma \varepsilon A\left(T^{4}-T_{0}^{4}\right)$$

(7) $$G_{r}=\sigma \varepsilon A\left(T+T_{0}\right)\left(T^{2}+T_{0}^{2}\right)$$

where *σ* is the Stefan–Boltzmann radiation constant, *ε* is the thermal emissivity of the suspended film, *T* is the equilibrium temperature of the thermal area, *T*_0_ is the ambient temperature, and *A* the area of the thermal area, respectively. The thermal radiation, however, is negligible here since the maximum temperature of this Pirani gauge is set below 120 °C.

In this work, we use the dilute gas heat transfer theory to calculate the thermal conductivity of the gas conduction [10]. Then,

(8) $$k=k_{0}{\left(1+2\left(\frac{2-a}{a}\right)\frac{l}{d}\cdot \frac{9.5}{6}\right)}^{-1}$$

where *k*_0_ is the thermal conductivity of the gas at atmospheric pressure (*k*_0_ = 25.6 × 10^−^^3^ W/(m·K)), *a* the energy adjustment coefficient (EAC) of the gas molecule between the two surfaces, and *l* the mean free path of gas molecules, respectively. For simplicity, we assume that the gas in the cavity is pure N_2_. The reason we use N_2_ is that the Pirani gauge requires different calibrations for different gases, as different gases have different EAC [11]. In this article, the environment in the test rig we built is N_2_. After encapsulation, if the vacuum environment is broken, the internal environment is also dominated by N_2_, and then *a* = 0.77 and *l·P* = 6.67 × 10^−^^3^ m·Pa, with *P* being the gas pressure.

In summary, the relationship between gaseous conduction *G_g_(P)* and air pressure *P* can be expressed as Equation (9).

(9) $$G_{g}\left(P\right)=k_{0}\frac{A}{d}{\left(1+\frac{33.74\times 10^{-5}\;m\cdot Pa}{Pd}\right)}^{-1}$$

## 3. Design and Simulation

### 3.1. Design of Structure

**Figure 2:**
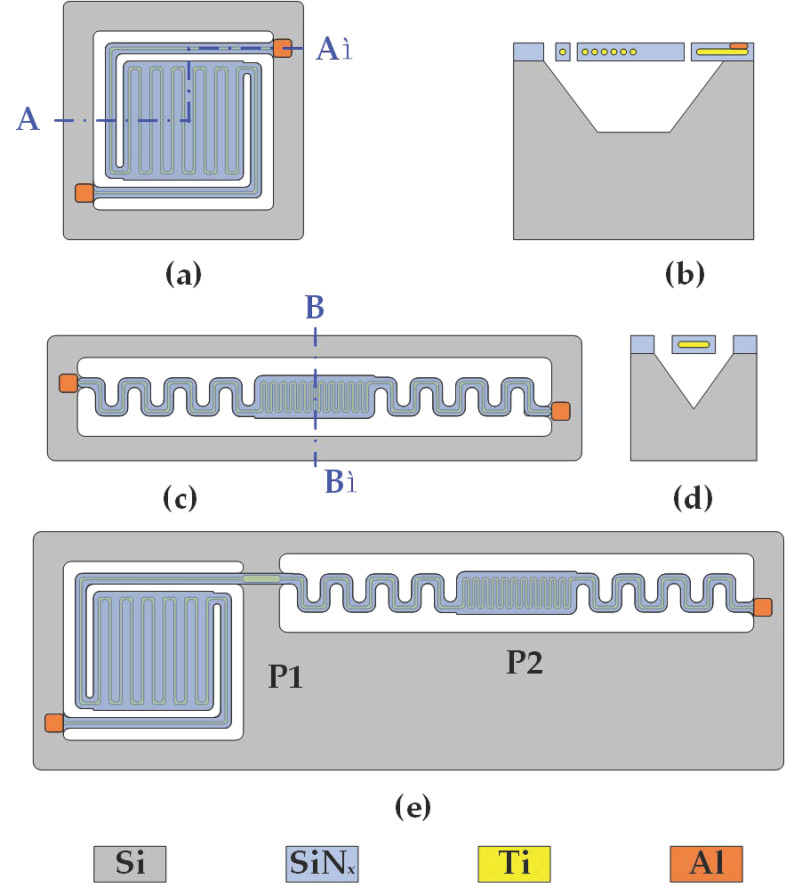
Designed structure of the Pirani gauge in this work. (**a**) Top structural view of P1; (**b**) A-A’ cross-sectional view of P1; (**c**) Top structural view of P2; (**d**) B-B’ cross-sectional view of P2; (**e**)Top structural view of composite-type MEMS Pirani gauge.

Conventional Pirani gauges can be classified into three categories based on their mechanical structure: micro-thermal plate type, micro-thermal bridge type, and micro-thermal gap type. The structure of the self-heating unit-getter has been designed and simulated in our previous study [7]. To complement our previous study, in this work, we chose the microheater plate as the thermal structure of the Pirani gauge so that it can be fabricated together with the microheater in Ref. [7]. As discussed in Section 2, Theoretical background, the range of measurement of the Pirani gauge is influenced by the size of the thermal area A and the size of the air gap d. In terms of structural design, this study referred to the ideas in Ref. [9]. The composite construction is superior because it allows one to tune the performances of the whole Pirani gauge by adjusting the sizes of the heating areas and the air gaps of two individual sensors. As shown in Figure 2, in this work, our Pirani gauge consists of P1, a Pirani gauge with a larger thermal area, and P2, a Pirani gauge with a smaller thermal area.

The structural design of the thermistor is essential for enhancing the performance of the Pirani gauge. However, the selection of the material for the structures of the dielectric layer and the thermistor layer is also important. The structure of the thermistor even needs to be optimized for different materials.

### 3.2. Materials

**Figure 3:**
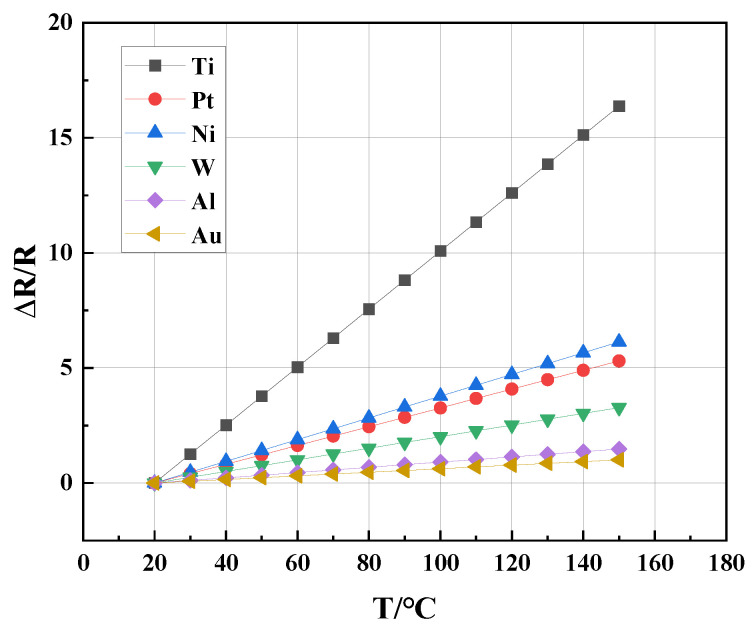
Change rate of resistance of several metals with temperature.

First, for the thermistor, this study compared the resistivity and temperature coefficient of resistance (TCR) of several commonly used metals, as shown in Table 1. The materials were placed over the same range of temperature variations in Figure 3. Platinum (Pt) is often used as a resistive material due to its resistance to high temperatures and corrosion. However, Pt is inferior to titanium (Ti) for a Pirani gauge because its change rate of specific resistance (∆R/R) with temperature is lower than Ti. The larger ∆R/R is, the larger the signal difference of a thermistor with temperature changes. Ti was then chosen as the material of the thermistor in this work. Since it is relatively easy for Ti to become corroded in the Pirani gauge fabricating process, it was protected by SiN_x_ when necessary. Ref. [12] indicates that Ti reacts easily with oxygen when in contact with hot air. For the natural oxide layer, the thickness is initially only 1 to 2 nm and will slowly thicken. It could reach 25 nm after four years. So, the brief contact with air has a negligible effect on this study.

In the MEMS field, the most common dielectric layers are SiO_2_ and SiN_x_. Some relevant properties of SiO_2_ and SiN_x_ are compared in Table 2, with some data from COMSOL Multiphysics. Since SiN_x_ has better mechanical properties and is more resistant to chemical corrosion, it is finally chosen as the dielectric layer material in this work.

### 3.3. Simulation

After having determined the materials for the Pirani gauge, a model for simulation in COMSOL Multiphysics was built with the geometric parameters in Table 3. In the simulation, the “electromagnetic thermal” multiphysics field was added for the coupling of the physical fields “current” and “heat conduction”. The thermal conductivity of gas heat conduction was calculated according to Equation (10),

(10) $$q=\frac{k}{d}\left(T-T_{0}\right)$$

where *q* is the heat flux coefficient.

#### 3.3.1. Voltage Input

**Figure 4:**
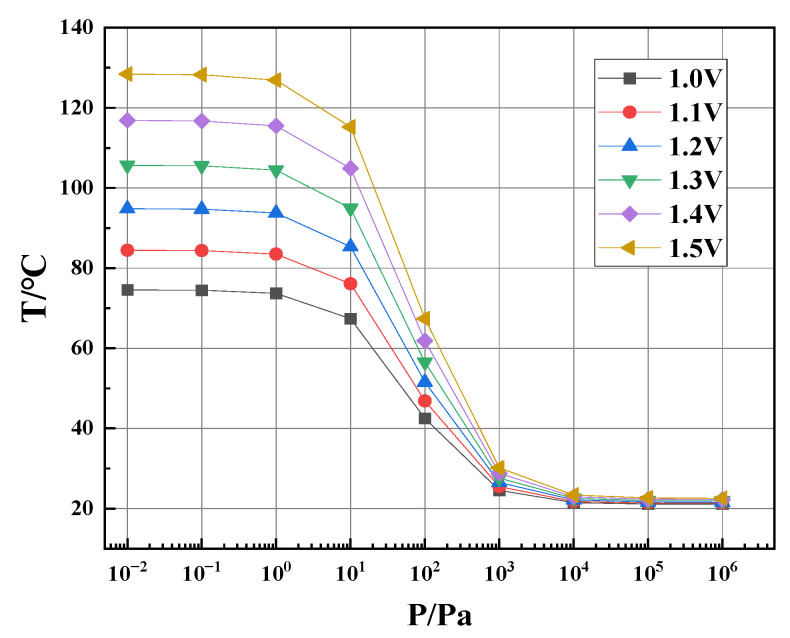
Simulated thermistor temperature as a function of different air pressures at different input voltages.

As shown in Figure 4, the input voltage to the thermistor is adjusted so that the range of temperature of the thermistor is large enough but less than 100 °C, while the maximum temperature is below 120 °C. In our previous paper, we have proven that the performance of the whole device is better than a single P1 or P2 [13]. So, the simulation results are from the whole device. Figure 4 suggests that the higher the input voltage, the higher the thermistor temperature and, therefore, the larger the change in the thermistor resistance. We set the temperature limit for such a consideration: If the thermistor is heated to a too-high temperature, plastic deformation may occur in the floating thermal area (also called the membrane) and may even lead to a permanent increase in the thermistor resistance, which means function failure of the Pirani gauge. Therefore, an input voltage of 1.2 V is selected at the beginning of the simulation.

#### 3.3.2. Dielectric Thickness

**Figure 5:**
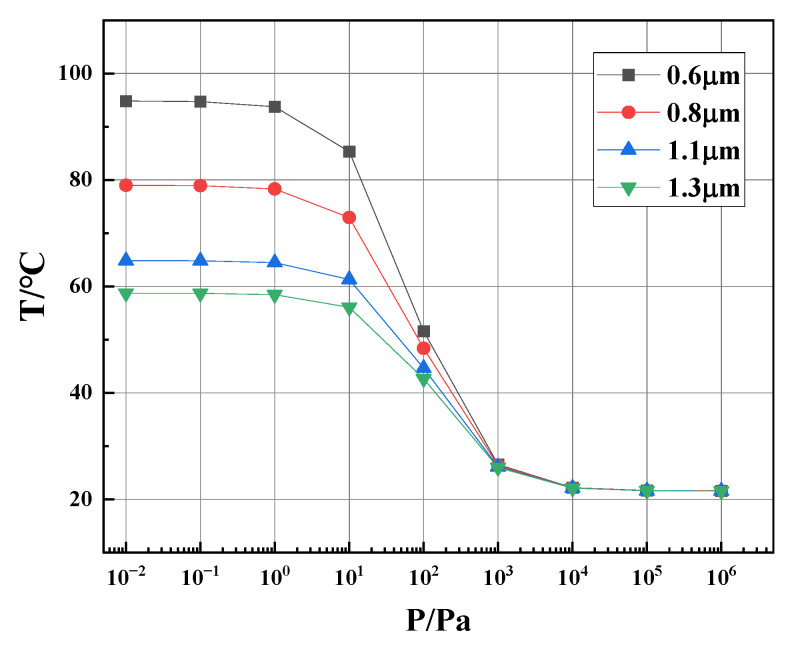
Simulated thermistor temperature as the function of air pressures for different dielectric thicknesses.

The heat conduction is determined with the geometrical parameters of the Pirani gauge device. We first optimized the thickness of the dielectric layers by simulation. In Figure 5, at an input of 1.2 V, it is found that the thinner the dielectric layer, the better the thermistor performance. On the other hand, if the dielectric layer is too thin, the mechanical strength of the membrane will get weak, which may lead to fabrication and usage problems. Therefore, in the following simulation, a thickness of 0.6 µm for the dielectric layer is initially used.

#### 3.3.3. Air Gap

It was shown that the air gap had no significant effect on the Pirani gauge performance at low pressure [9], where the mean free path of the gas molecules was larger than the size of the air gap. However, at high pressures, the air gap size might get larger than the mean free path of the gas molecules and affect the Pirani gauge performance according to the pressure. In order to extend the measurable range of the Pirani gauge at the high-pressure end, the P2 air gap was designed to be smaller than that of P1 in this work: the initial air gaps in the simulation were 70 µm for P1 and 40 µm for P2. Experimentally, an average etching rate of Si in the <100> crystal direction is 0.2~0.3 µm/min in 30% KOH at 80℃. The air gap depth *d* was then controlled by the etching time of the cavity in KOH and then confirmed after etching.

## 4. Fabrication Process

**Figure 6:**
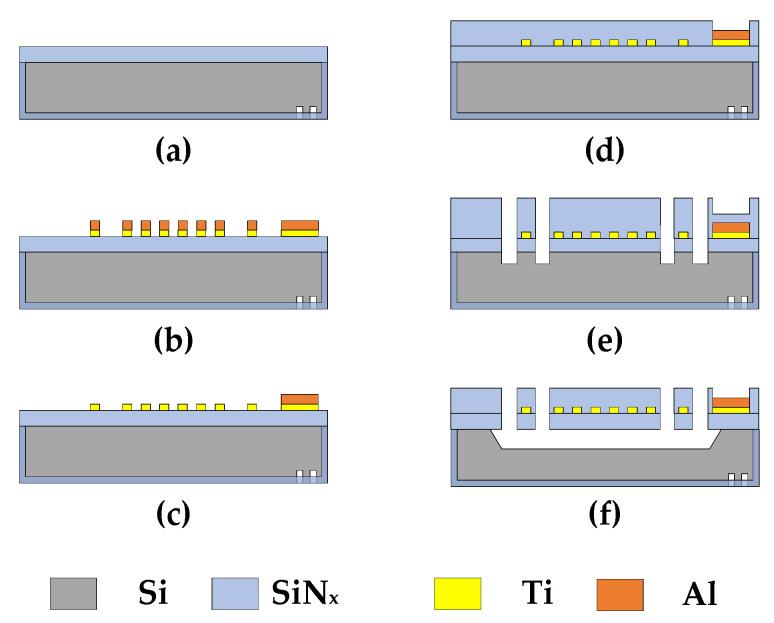
Main steps of the fabrication flow of the designed MEMS Pirani gauge. (**a**) Double-sided deposition of SiN_x_; (**b**) Metal layer pattern formation; (**c**) Wet etching of Al to form the pads; (**d**) Deposition and dry etching of SiN_x_; (**e**) Dry etching of SiN_x_ and Si to form the cavity release windows; (**f**) Cavity formation by KOH etching of Si and pad exposure by blanket etching of SiN_x_.

The designed Pirani gauge was then fabricated with an 8-inch MEMS process. Figure 6 shows the main fabricating steps for simplicity. First, in Figure 6a, SiN_x_ films were deposited on both sides of an 8-inch silicon wafer. In Figure 6b, titanium and aluminum films were deposited and dry-etched consequently on the front side of the silicon wafer as the thermistor and electrodes, respectively. In Figure 6c, the aluminum pads were finally defined after wet etching. In Figure 6d, the deposition of SiN_x_ protective film on the thermistor and pad area open was carried out. In Figure 6e, a SiN_x_ film was deposited to protect the pad area, and the cavity release windows were opened. In Figure 6f, a KOH etch via the cavity release windows was performed to form the cavity underneath the thermistor, and finally, a SiN_x_ blanket etch followed to expose the pad area.

**Figure 7:**
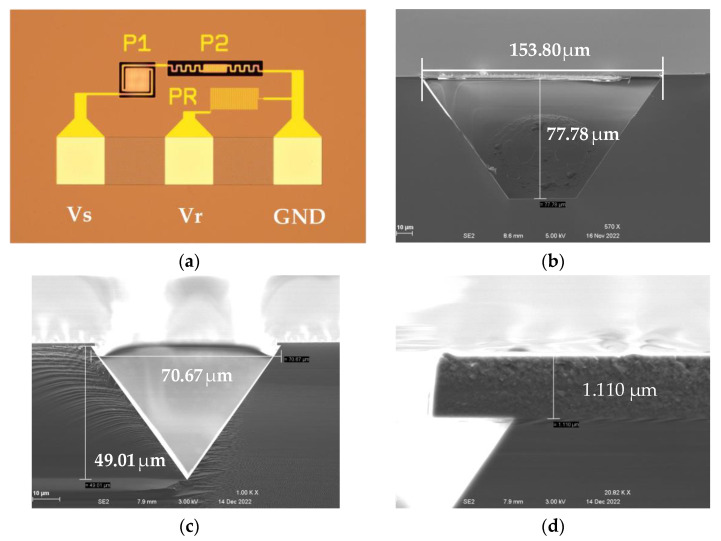
Micrographs of a fabricated MEMS Pirani gauge. (**a**) Top optical microscopic view of the whole device; (**b**) Cross-sectional SEM view of P1 Gauge; (**c**) Cross-sectional SEM view of P2 Gauge; (**d**) Cross-sectional SEM view of the dielectric layer.

The photograph of a fabricated Pirani gauge device is shown in Figure 7a, where P1 and P2 stand for Gauge 1 and Gauge 2, respectively, while PR stands for the reference resistance R_r_. Various cross-sections of the Pirani gauge device are observed using SEM and shown in Figure 7b–d.

## 5. Pirani Gauge Performance Evaluation

**Figure 8:**
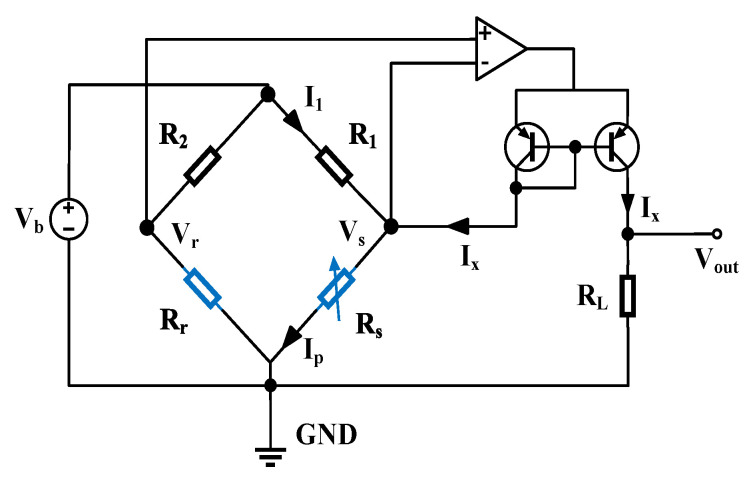
Constant-bias Wheatstone bridge circuit for the Pirani gauge operation [14].

By using the thermistor temperature obtained from the simulation, the thermistor resistance values can be calculated. The resistance values were then assigned to Rs in the NI Multisim simulation of the circuit shown in Figure 8 [14]. Following Ref. [14], the reference resistor R_r_ was made of the same material as R_s_ and was formed directly on the silicon substrate so that its temperature remained the same as the environment. By doing this, we could reduce the influence of the ambient temperature on the whole sensor due to the usage of the Wheatstone bridge circuit. The circuit parameters set in this simulation were V_b_ = 2.4 V, R_1_ = R_2_ =R_L_ = 10 kΩ, and R_r_ = 10 kΩ at 20 °C.

**Figure 9:**
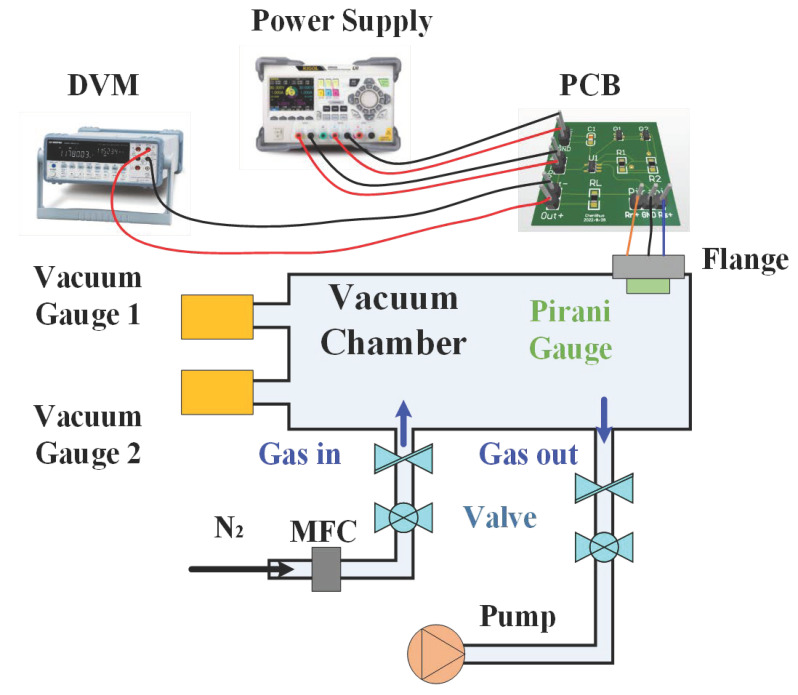
A platform built to carry out the Pirani gauge performance evaluation.

In order to carry out the Pirani gauge performance evaluation, a platform was built, as shown in Figure 9. During the measurements, pure N_2_ gas was used to control the pressure (or vacuum level) in the chamber. The pressure level was adjusted by controlling the inlet and outlet valves. A pin-hole plug-and-play adapter was also fabricated to nestle in the flange of the vacuum chamber. The connection was achieved between the internal Pirani gauge device and an external circuit. The external circuit included a printed circuit board (PCB), a power supply, and a digital voltmeter (DVM). To read the pressure in the chamber, we used two vacuum gauges: an MKS Baratron capacitance manometer (627D12TDC1B) for low-pressure measurement and an Edwards active Pirani gauge (APGX-L-NW16 ST/ST) for high-pressure measurement.

During the measurements, every output from the Pirani gauge was read after the chamber pressure had stabilized at the determined value for 10 s. The maximum and minimum values of the output displayed on the DVM were recorded. The output variations (the difference between the maximum and minimum values) remained less than 0.1 mV during the measurements. The actual points of measurement range from 6.6 × 10^−2^ to 1.12 × 10^5^ Pa, with the upper and lower ends limited by the system, not the Pirani gauge.

**Figure 10:**
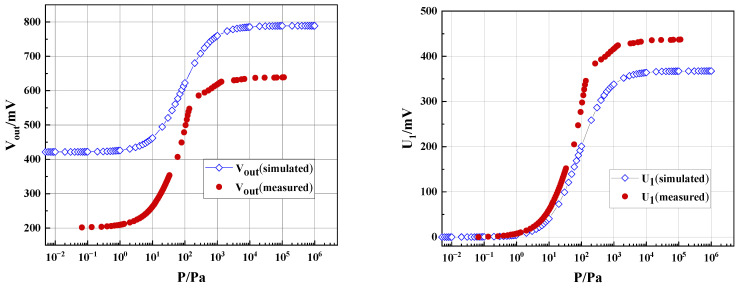

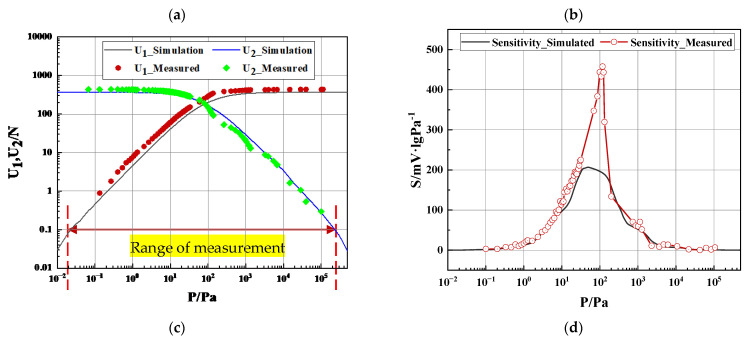
Performance of the Pirani gauge (experiment and simulation)**.** (**a**) Raw data of circuit outputs together with the simulation results. (**b**) U_1_ curves. (**c**) Analysis of ranges of measurement. (**d**) Analysis of sensitivity.

The measured raw data of the Pirani gauge output are shown in Figure 10a, together with the results from the simulation. In Figure 10a, there exists a discrepancy between the measurement and simulation results. The discrepancy may mainly originate from the derivations of geometrical parameters of the thermal area and supporting beams and the resistance of the thermistor between the real device and the simulation. In fact, we found great resistance value changes from the simulation to the experiment: R_s_ from 5.88 kΩ to 10.09 kΩ at 20 °C, Rr from 10 kΩ to 17.52 kΩ. From Figure 7, we also found that the air gaps changed from 70 µm to 77.78 µm for P1 and from 40 µm to 49.01 µm for P2, from design to experiment; the thickness of the dielectric layer was increased deliberately from 0.6 µm (initial value for the simulation) to 1.11 µm for mechanical strength consideration.

In order to facilitate statistical calculations and extract more useful information from the data in Figure 10a, we performed data analyses. Here we introduced *U_1_* and *U_2_* [15], with

(11) $$U_{1}=U-U_{min}$$

(12) $$U_{2}=U_{max}-U$$

where *U* is the actual output voltage (Also *V_out_* in Figure 8), *U_max_* is the maximum output voltage, and *U_min_* is the minimum output voltage, respectively. From the simulation, the voltage resolution *N* can be derived from the upper limit *P_h_* and lower limit *P_l_* of the pressure measurement. Referring to previous studies [10,15,16], the voltage resolution *N* = 0.1 mV was chosen in this research. The equation for the average sensitivity *S_m_* calculation is shown in Equation (13) [16]:

(13) $$S_{m}=\frac{U_{max}-U_{min}}{lg^{P_{h}}-lg^{P_{l}}}$$

Using the simulation, when the voltage sensitivity is set to 0.1 mV, a range of measurement of 2 × 10^−2^ to 2 × 10^5^ Pa and an average sensitivity of 52.4 mV/lgPa can be obtained. An average sensitivity of 52.4 mV/lgPa means that for each order of magnitude change in pressure, the output voltage of the Pirani gauge will change by 52.4 mV.

Figure 10b shows *U*_1_ as the function of pressure. Despite the large discrepancy in Figure 10a, the trends of the two curves from measurement and simulation in Figure 10b are quite consistent, suggesting that the simulation results are good guidance for device design. As shown in Figure 10c, the actual range of measurement of pressure is close to the simulated values of 2 × 10^−2^ to 2 × 10^5^ Pa. As shown in Figure 10d, the actual average sensitivity is much larger than the simulated value of 52.4 mV/lgPa, with the highest sensitivity up to 457.6 mV/lgPa. It has good linearity at 10–1000 Pa, and the average sensitivity in this range is 179.2 mV/lgPa, which is also better than the simulation result of 148.8 mV/lgPa.

## 6. Conclusions

This study proposed a composite-type MEMS Pirani gauge of a wide range and high accuracy. High TCR material Ti is used as the thermistor, and SiN_x_ is used as the dielectric layer material. This Pirani gauge was fabricated based on simulation results. In comparison with the simulation curve, it can be found that the fabricated Pirani gauge has a range of measurement close to the simulation value of 2 × 10^−2^ to 2 × 10^5^ Pa. Meanwhile, the average sensitivity is much larger than the designed value of 52.4 mV/lgPa. The resolution is 0.1 mV. The Pirani gauge also has the advantages of simple structure, easy fabrication, and easy compatibility with CMOS processes. Moreover, the Pirani gauge could be integrated with a MEMS device within the same vacuum cavity by wafer bonding to monitor the gas pressure therein in real-time.

## Figures and Tables

**Table 1:** Resistivity and TCR of several metallic materials.

Materials	Resistivity (µΩ·m)	TCR (%/K)
Ti	0.420	0.300
Pt	0.106	0.385
Ni	0.068	0.690
W	0.056	0.450
Al	0.027	0.429
Au	0.024	0.324

**Table 2:** Physical properties of SiO_2_ and SiN_x_.

Materials	Heat Capacity	Young Modulus	Poisson’s Ratio	Etch Rate in KOH
SiO_2_	730 J/(kg·K)	7 × 10^4^ MPa	0.17	10~30 nm/min
SiN_x_	700 J/(kg·K)	2.5 × 10^5^ MPa	0.23	1~3 nm/min

**Table 3:** Design parameters.

Parameters	P1	P2
Thermal area (µm × µm)	100 × 100	100 × 36
Cantilever beam (µm × µm × µm)	500 × 9 × 0.6	630 × 8 × 0.6
Cavity opening (µm × µm)	150 × 150	395 × 66
Turn number of thermistor	11	23
Resistance cross-section (µm × µm)	2 × 0.1	2 × 0.1
Air gap (µm)	60	46

## Data Availability

Not applicable.
